# Supplementary material for: Structural mechanisms of the human cardiac sodium-calcium exchanger NCX1
Source: Nat Commun. 2023 Oct 4;14:6181. doi: 10.1038/s41467-023-41885-4 (PMC10550945; doi:10.1038/s41467-023-41885-4)
Supplement: Supplementary file 1 — Supplementary Information [file 41467_2023_41885_MOESM1_ESM.pdf]

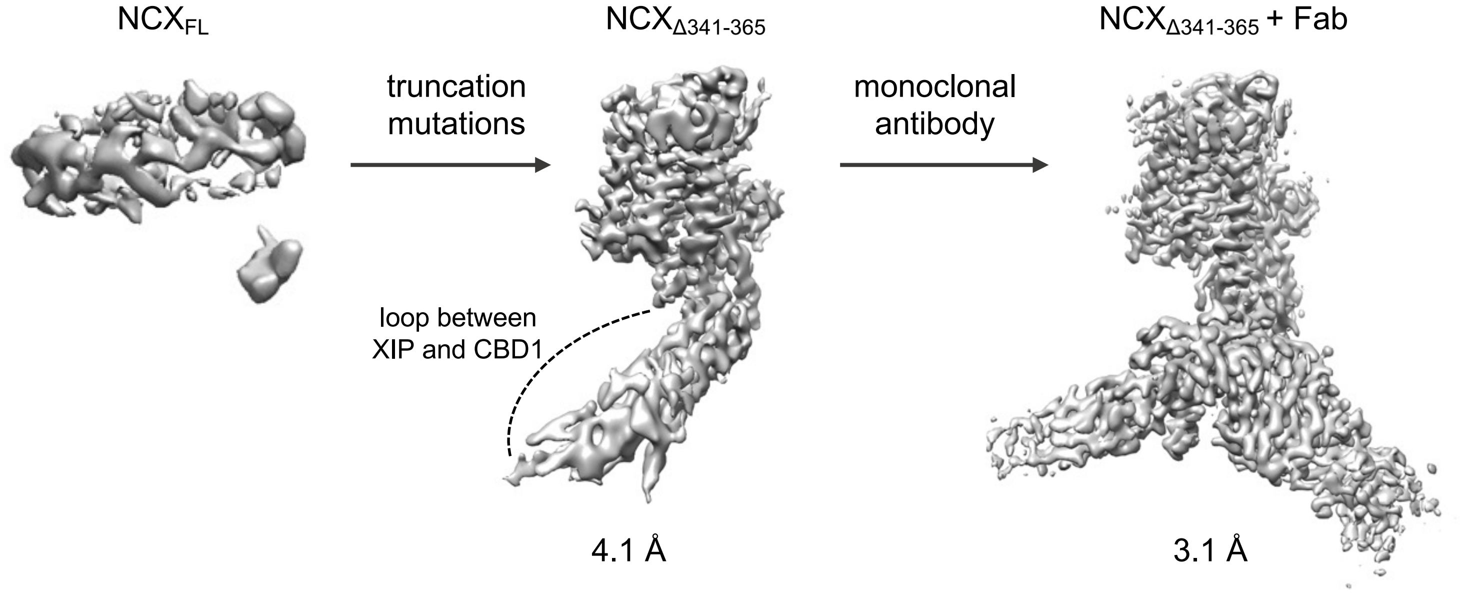

Supplementary Fig. 1 Constructs for structure determination of human cardiac NCX1. Shown are 3D reconstructions of particles from various NCX1 constructs. 3D reconstruction of the full-length NCX1 yielded a poorly resolved map (left), likely because of the small size of the exchanger protein along with a highly mobile cytosolic domain. Deletion of residues 341-365 at the loop between XIP and CBD1 improves the stability of the exchange and yields a 4.1 Å map from 3D reconstruction (middle). The binding of Fab further stabilizes the exchanger and yields a 3.1 Å map from 3D reconstruction (right).

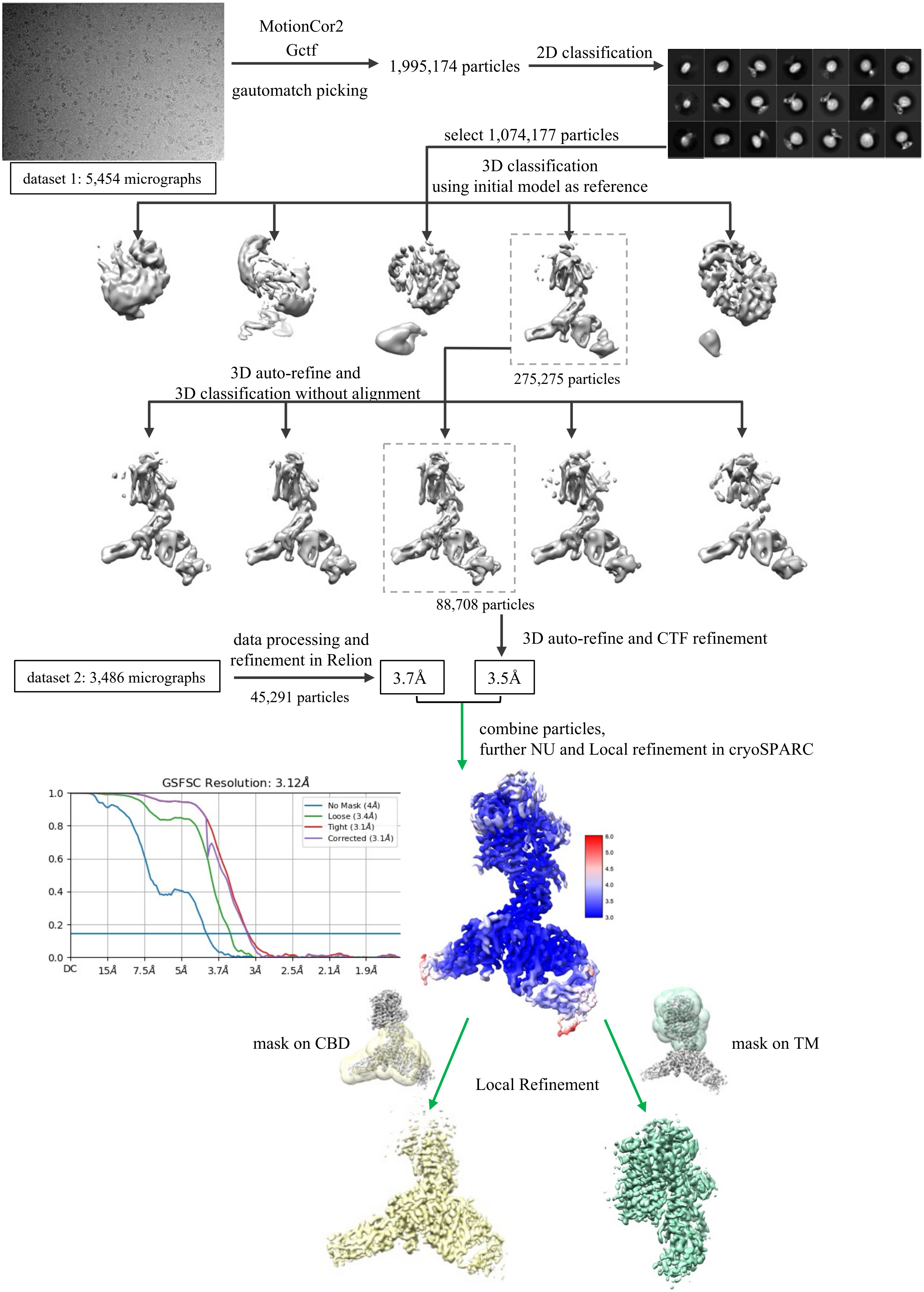

Supplementary Fig. 2 Cryo-EM data processing scheme of the apo, inactivated NCX1.

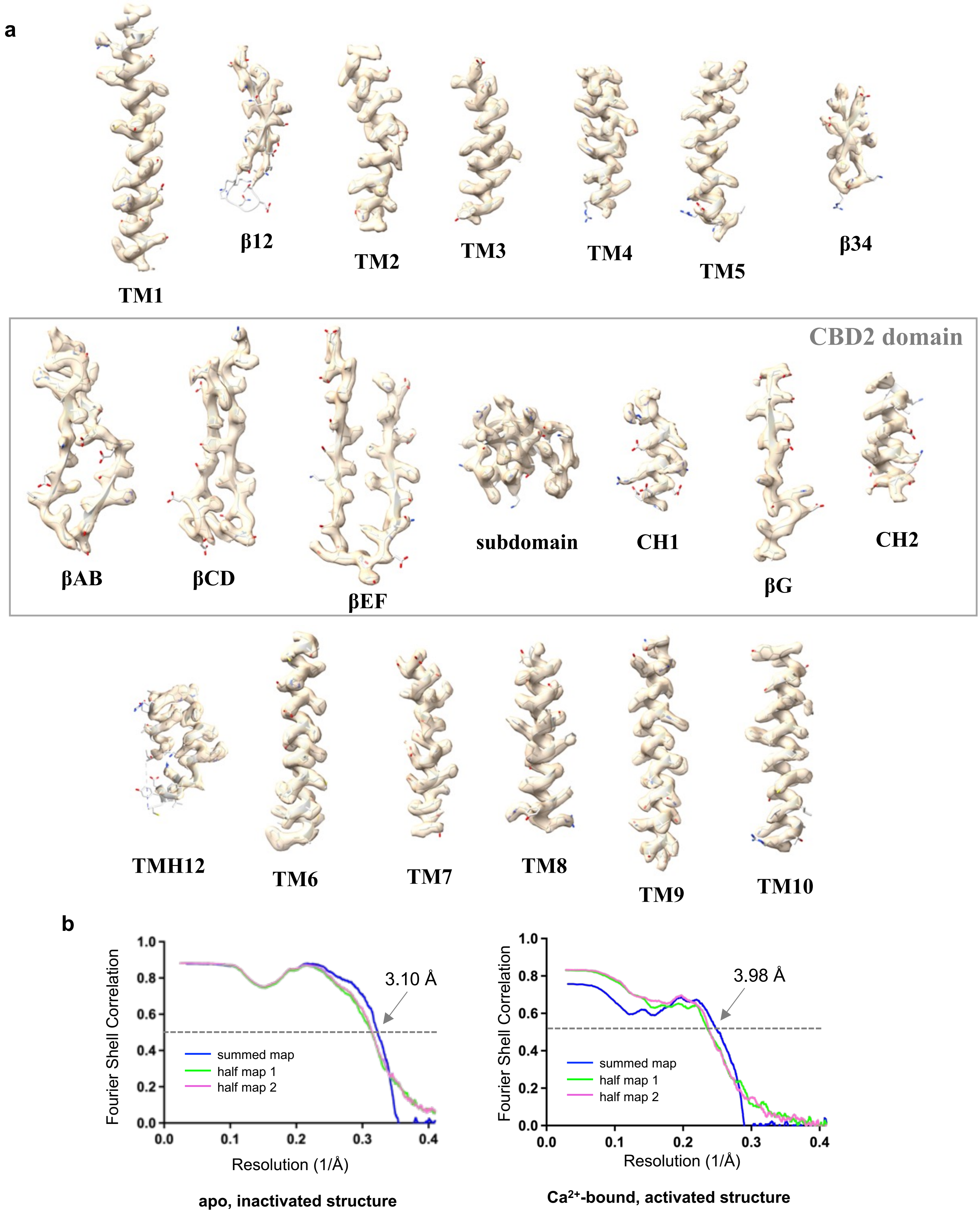

Supplementary Fig. 3 Sample density maps of human NCX1. a, Density maps of the apo, inactive NCX1 structure contoured at the threshold level of 0.71 using the ChimeraX software. **b,** The Fourier shell correlation (FSC) curves for cross-validation between the maps and the models. Curves for model versus summed map in blue (sum), for model versus half map in green, and for model versus half map not used for refinement in pink.

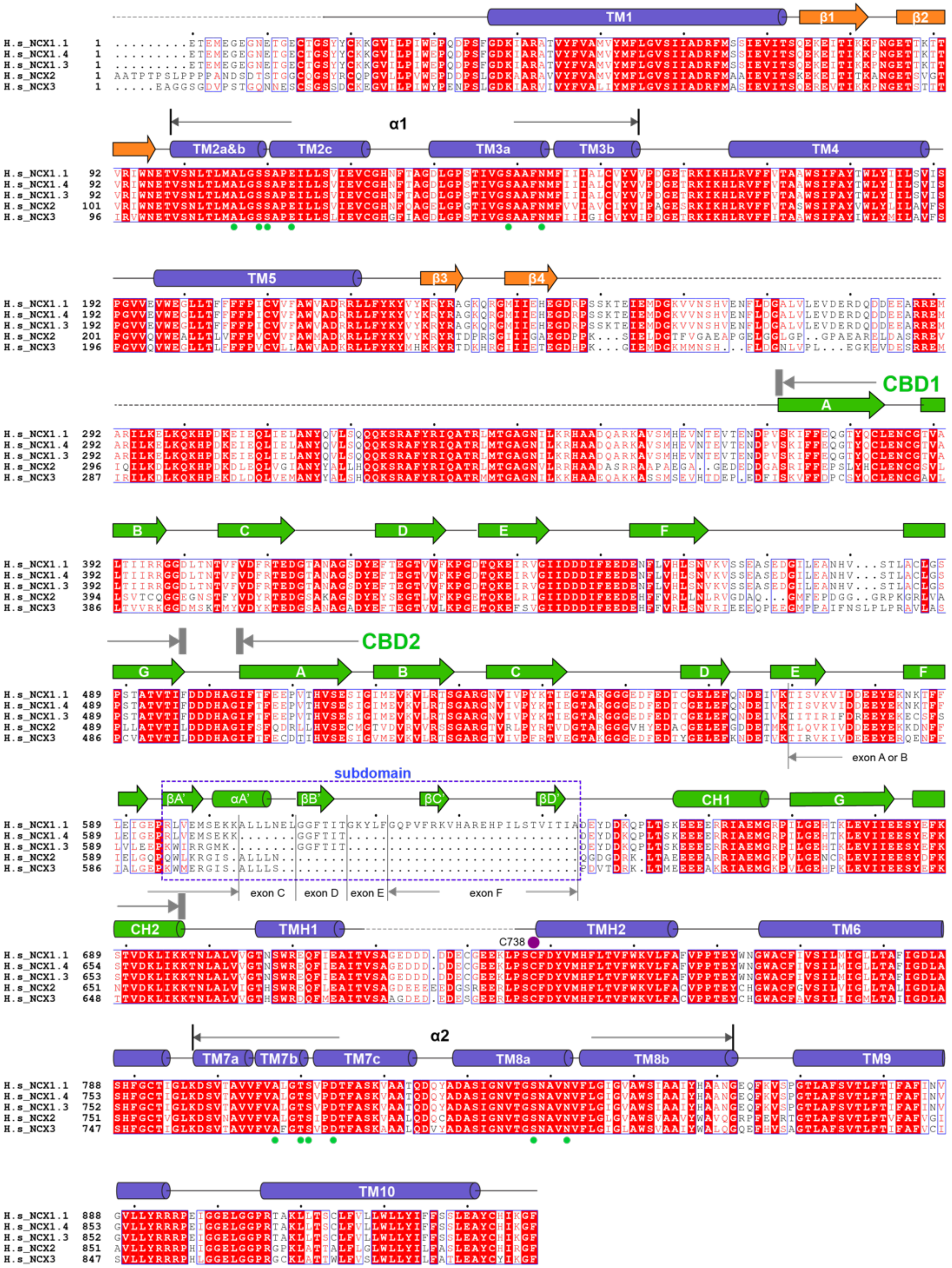

Supplementary Fig. 4 Sequence alignment and secondary structure assignments of NCX. Residues are numbered without signal peptide. Key residues for TM ion binding sites are marked by green dots. NCX1.1 (exon ACDEF), NP_066920.1; NCX1.4 (exon AD), NP_001338421.1; NCX1.3 (exon BD), NP_001338423.1; NCX2, NP_055878.1; NCX3, NP_892114.1.

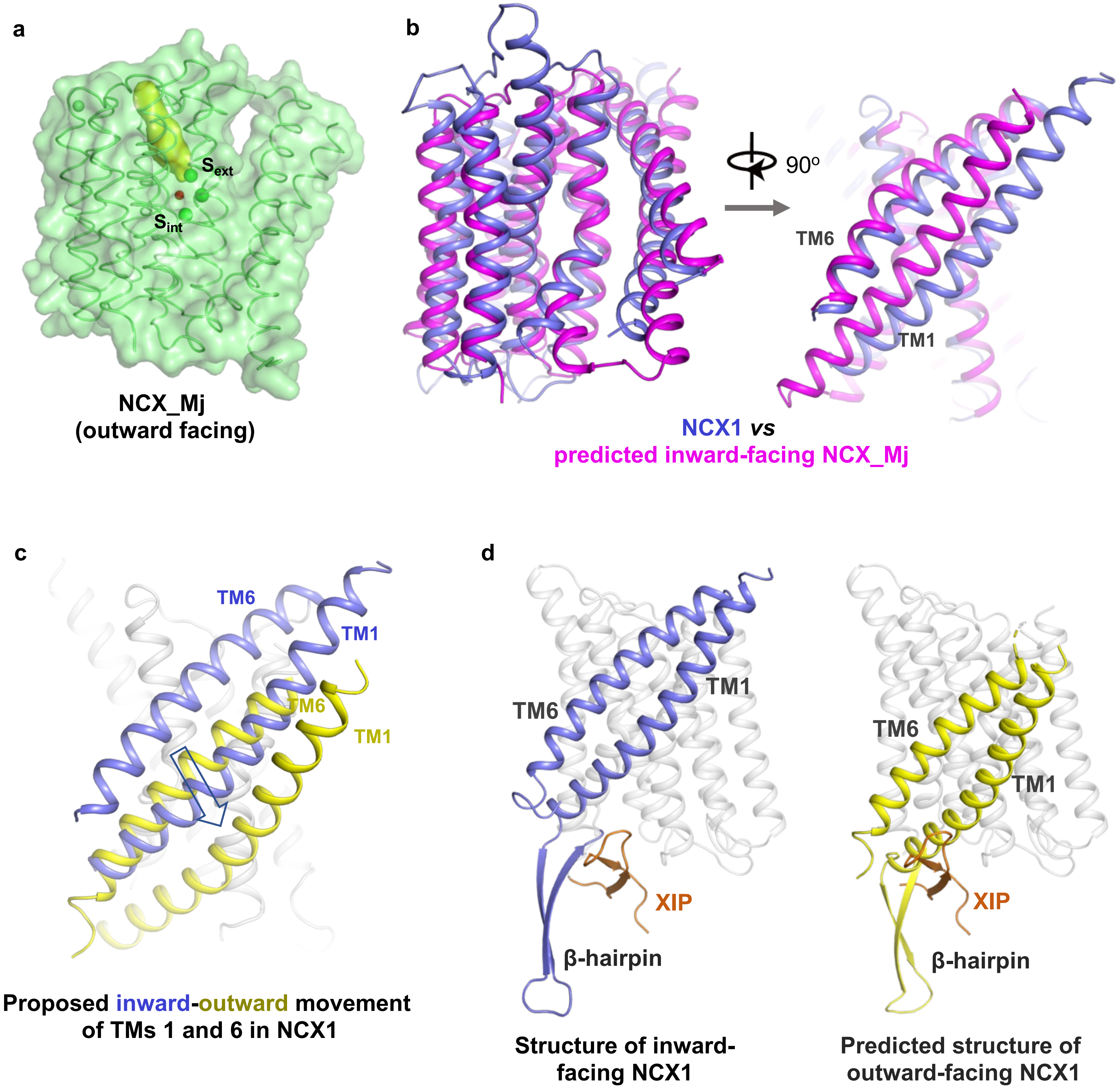

Supplementary Fig. 5 Proposed inward-outward conformational changes in NCX1. a, Surface-rendered outward-facing NCX_Mj structure showing the external passage (colored in yellow) for ion access to S_{ext}. **b,** Structural comparison between the NCX1 TM domain and the predicted inward-facing NCX_Mj from the previous study (Liao et al., 2012). **c,** Predicted inward-outward conformational changes in NCX1. The outward-facing NCX1 was generated based on the structure homology between the two halves of the TM domain as described previously (Liao et al., 2012) as well as the structural comparison between the inward-facing NCX1 and outward-facing NCX_Mj. **d,** The predicted movement of β-hairpin along with TMs 1 and 6 from inward (blue) to outward (yellow) conformation would cause a direct collision with XIP.

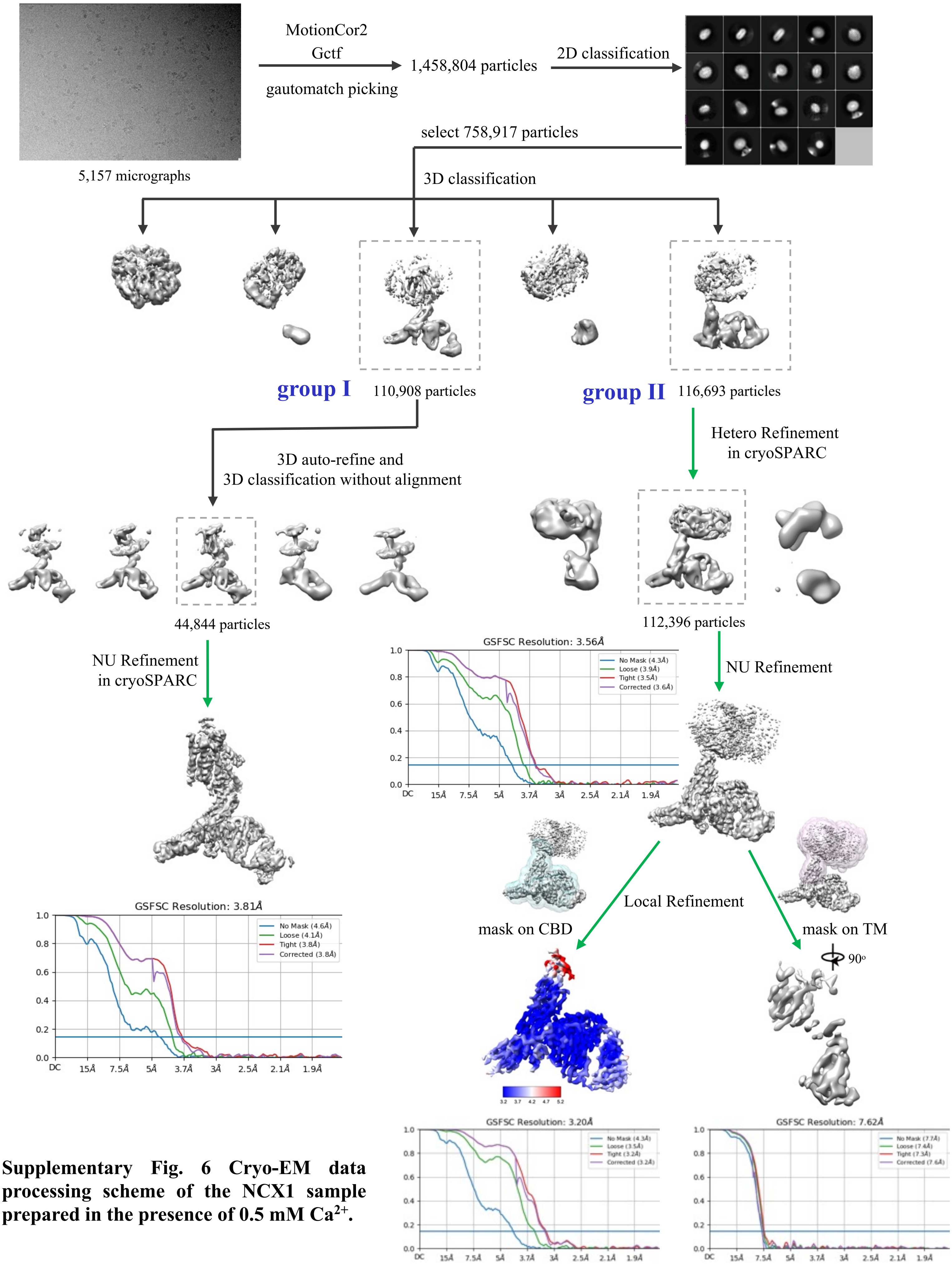

Supplementary Fig. 6 Cryo-EM data processing scheme of the NCX1 sample prepared in the presence of 0.5 mM Ca^{2+}.

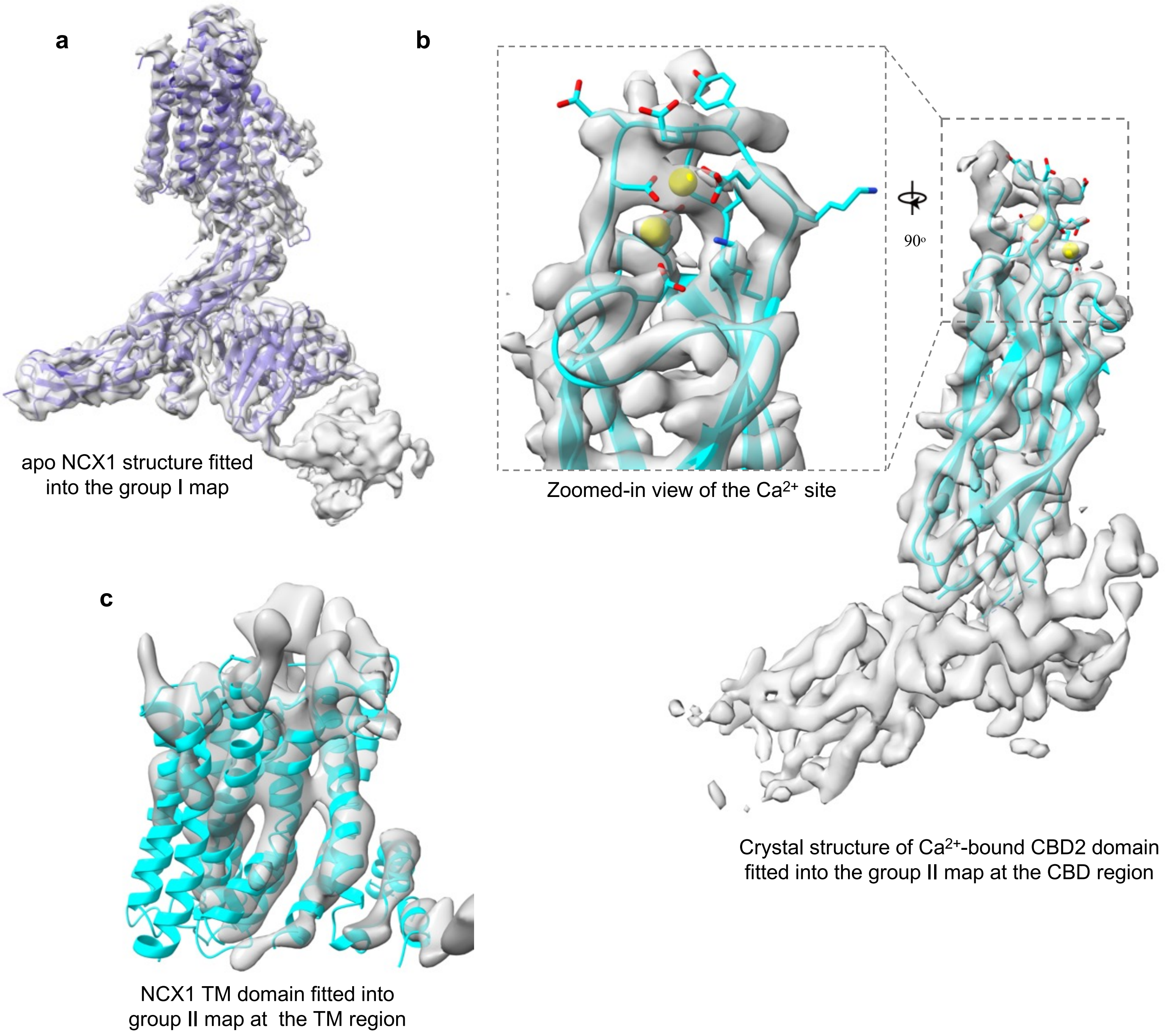

Supplementary Fig. 7 Modeling of the group I & II structures of NCX1 prepared in the presence of 0.5 mM Ca^{2+}. a, Docking the apo NCX1 structure (8SGJ) into the EM map from group I particles. **b,** Docking the crystal structure of Ca^{2+}-bound CBD2 (PDB: 2QVM) (Besserer et al., 2007) into the EM map from group II particles with a zoomed-in view of the Ca^{2+} sites. **c,** Docking the NCX1 TM domain structure into the low-resolution EM map from group II particles after focused refinement at the TM region.

**Sample preparation conditions**	25 mM Hepes pH 7.4, 200 mM NaCl	25 mM Hepes pH 7.4, 200 mM NaCl, 0.5 mM Ca^{2+}	
	**apo, inactivated state** (EMD-40457, PDB 8SGJ)	**apo inactivated state, group I** (EMD-40460)	**Ca^{2+}-bound activated state, group II** (EMD-40467, PDB 8SGT)
**Data collection and processing**			
Magnification	105,000	105,000	105,000
Voltage (kV)	300	300	300
Electron exposure (e^{-}/Å^2)	60	60	60
Defocus range (μm)	-0.9 - -2.2	-0.9 - -2.2	-0.9 - -2.2
Pixel size (Å)	0.83	0.83	0.83
Symmetry imposed	C1	C1	C1
Initial particle images (no.)	1,598,642	758,917	758,917
Final particle images (no.)	133,999	44,844	112,396
Map resolution (Å)	3.12	3.81	3.56
FSC threshold	0.143	0.143	0.143
**Refinement**			
Initial model used (PDB code)	8SGI	8SGI (the structure model can be directly fitted into the map without refinement	8SGI, 2QVM
Model resolution (Å)	3.36		3.98
FSC threshold	0.5		0.5
Model composition			
Non-hydrogen atoms	7667		7119
Protein residues	982		918
Ligands	3: Na<br>6: Ca<br>1: H_2O		6: Ca
B factors (Å^2)			
Protein	46.64		97.41
Ligands	38.18		88.92
R.m.s. deviations			
Bond lengths (Å)	0.004		0.005
Bond angles (°)	0.702		0.754
Validation			
MolProbity score	1.40		1.28
Clashscore	5.32		4.53
Poor rotamers (%)	0		0
Ramachandran plot			
Favored (%)	97.41		97.78
Allowed (%)	2.59		2.22
Disallowed (%)	0		0

Supplementary Table 1. Cryo-EM data collection and model statistics.
