# Supplementary material for: Structural mechanisms of the human cardiac sodium-calcium exchanger NCX1
Source: Nat Commun. 2023 Oct 4;14:6181. doi: 10.1038/s41467-023-41885-4 (PMC10550945; doi:10.1038/s41467-023-41885-4)
Supplement: Supplementary file 3 — Description of Additional Supplementary Files [file 41467_2023_41885_MOESM3_ESM.pdf]

Description of Additional Supplementary Files

File Name: Supplementary Movie 1
Description: The rotation of the NCX1 structural model.

File Name: Supplementary Movie 2
Description: The proposed inward-outward transition at TMs 1 and 6 along with β-hairpin as shown in Supplementary Fig. 5d, indicating the block of this movement by XIP when the exchanger is in the inward-facing, inactivated state.

File Name: Supplementary Movie 3
Description: Conformational changes at CBD2 from apo to Ca2+-bound states as depicted in Fig. 4a.

File Name: Supplementary Movie 4
Description: The morph of the NCX1 structures from the inactivated state with apo CBD2 to an activated state with Ca2+-bound CBD2 as shown in Fig. 4d. The βhub is disassembled in the Ca2+- activated state.
