# Supplementary material for: Structural mechanisms of the human cardiac sodium-calcium exchanger NCX1
Source: Nat Commun. 2023 Oct 4;14:6181. doi: 10.1038/s41467-023-41885-4 (PMC10550945; doi:10.1038/s41467-023-41885-4)
Supplement: Supplementary file 8 — Reporting Summary [file 41467_2023_41885_MOESM8_ESM.pdf]

nature portfolio

Corresponding author(s): Youxing Jiang

Last updated by author(s): Sep 18, 2023

Reporting Summary

Nature Portfolio wishes to improve the reproducibility of the work that we publish. This form provides structure for consistency and transparency in reporting. For further information on Nature Portfolio policies, see our Editorial Policies and the Editorial Policy Checklist.

Statistics

For all statistical analyses, confirm that the following items are present in the figure legend, table legend, main text, or Methods section.

n/a	Confirmed	
☐	☒	The exact sample size (*n*) for each experimental group/condition, given as a discrete number and unit of measurement
☒	☐	A statement on whether measurements were taken from distinct samples or whether the same sample was measured repeatedly
☐	☒	The statistical test(s) used AND whether they are one- or two-sided<br>*Only common tests should be described solely by name; describe more complex techniques in the Methods section.*
☒	☐	A description of all covariates tested
☒	☐	A description of any assumptions or corrections, such as tests of normality and adjustment for multiple comparisons
☐	☒	A full description of the statistical parameters including central tendency (e.g. means) or other basic estimates (e.g. regression coefficient) AND variation (e.g. standard deviation) or associated estimates of uncertainty (e.g. confidence intervals)
☐	☒	For null hypothesis testing, the test statistic (e.g. *F*, *t*, *r*) with confidence intervals, effect sizes, degrees of freedom and *P* value noted<br>*Give P values as exact values whenever suitable.*
☒	☐	For Bayesian analysis, information on the choice of priors and Markov chain Monte Carlo settings
☒	☐	For hierarchical and complex designs, identification of the appropriate level for tests and full reporting of outcomes
☒	☐	Estimates of effect sizes (e.g. Cohen's *d*, Pearson's *r*), indicating how they were calculated

Our web collection on statistics for biologists contains articles on many of the points above.

Software and code

Policy information about availability of computer code

Data collection	SerialEM 3.8
Data analysis	Relion3.1, Coot 0.8.9, Phenix 1.19.2, Pymol 2.4.2, Chimera 1.13, ChimeraX 1.4, CryoSPARC 3.3.0, MotionCor2 1.2.1, Graphpad Prism 8

For manuscripts utilizing custom algorithms or software that are central to the research but not yet described in published literature, software must be made available to editors and reviewers. We strongly encourage code deposition in a community repository (e.g. GitHub). See the Nature Portfolio guidelines for submitting code & software for further information.

Data

Policy information about availability of data

All manuscripts must include a data availability statement. This statement should provide the following information, where applicable:

- Accession codes, unique identifiers, or web links for publicly available datasets
- A description of any restrictions on data availability
- For clinical datasets or third party data, please ensure that the statement adheres to our policy

The cryo-EM density maps of the human NCX1 have been deposited in the Electron Microscopy Data Bank under accession numbers EMD-40457 for the apo inactivated state and EMD-40460 and EMD-40467 for the group I and II structures in the presence of 0.5 mM Ca2+, respectively. Atomic coordinates have been deposited in the Protein Data Bank under accession numbers 8SGJ for the apo inactivated structure and 8SGT for the group II Ca2+-bound activated structure.

Research involving human participants, their data, or biological material

Policy information about studies with human participants or human data. See also policy information about sex, gender (identity/presentation), and sexual orientation and race, ethnicity and racism.

Reporting on sex and gender	N/A
Reporting on race, ethnicity, or other socially relevant groupings	N/A
Population characteristics	N/A
Recruitment	N/A
Ethics oversight	N/A

Note that full information on the approval of the study protocol must also be provided in the manuscript.

Field-specific reporting

Please select the one below that is the best fit for your research. If you are not sure, read the appropriate sections before making your selection.

☒ Life sciences ☐ Behavioural & social sciences ☐ Ecological, evolutionary & environmental sciences

For a reference copy of the document with all sections, see nature.com/documents/nr-reporting-summary-flat.pdf

Life sciences study design

All studies must disclose on these points even when the disclosure is negative.

Sample size	The data size for cryo-EM was determined by the availability of the microscope time and the particle density on the grids. Sufficient cryo-EM datas were collected to achieve the reported resolution of map, which is sufficient for model building.
Data exclusions	CryoEM data processing involved removing poor-quality or damaged particles to achieve high resolution maps through pre-established standard data classification procedures.
Replication	For functional analysis,each experiment was reproduced at least two times on separate occasions. Experimental findings were reliably reproduced.
Randomization	Randomization was not necessary as the independent variables to be tested were sufficient for the functional interpretations within this study. i.e. WT vs mutant current recordings
Blinding	All experiments are not blind. Blinding is not necessary for the purposes of structural determination. For functional analysis, blinding was not necessary due to the quantitative nature of the experiment.

Reporting for specific materials, systems and methods

We require information from authors about some types of materials, experimental systems and methods used in many studies. Here, indicate whether each material, system or method listed is relevant to your study. If you are not sure if a list item applies to your research, read the appropriate section before selecting a response.

Materials & experimental systems

n/a	Involved in the study	
☐	☒	Antibodies
☐	☒	Eukaryotic cell lines
☒	☐	Palaeontology and archaeology
☒	☐	Animals and other organisms
☒	☐	Clinical data
☒	☐	Dual use research of concern
☒	☐	Plants

Methods

n/a	Involved in the study	
☒	☐	ChIP-seq
☒	☐	Flow cytometry
☒	☐	MRI-based neuroimaging

Antibodies

Antibodies used	Human NCX1 Fab 2E4 were raised by Vector and Gene Therapy Institute (VGTI) at Oregon Health & Science University (OHSU) using purified human NCX1 by us.
Validation	Fab 2E4 binding to human NCX1 was shown by the co-migration of the Fab and the transporter on the size exclusion column, and the Fab density in the cryo-EM map of NCX1-Fab 2E4 complex.

Eukaryotic cell lines

Policy information about cell lines and Sex and Gender in Research

Cell line source(s)	Human embryonic kidney (HEK293 GnTi-) purchased from ATCC and Sf9 insect cells purchased from Thermo Fisher Scientific.
Authentication	No further authentication was performed for commercially available cell lines.
Mycoplasma contamination	Cell lines were not tested for mycoplasma contamination.
Commonly misidentified lines (See ICLAC register)	None.
